# Evidence-Based Consensus on the Diagnosis and Treatment of Advanced HER2-Positive and HER2-Low Breast Cancer in Colombia

**DOI:** 10.3390/jcm15020514

**Published:** 2026-01-08

**Authors:** Mauricio Lema, Luz F. Sua, Abrahám José Hernández Blanquisett, William Mantilla, Marc Edy Pierre, Hernán Carranza, Julio Ricardo Zuluaga Peña, Aura Victoria Gutiérrez Raba, Sandra Ximena Franco

**Affiliations:** 1 Oncology Clinic Astorga, Medellín 050021, Colombia; 2 Faculty of Health Sciences, Universidad Icesi, Cali 760031, Colombia; lufer24@hotmail.com; 3 Department of Pathology and Laboratory Medicine, Fundación Valle del Lili, Cali 760031, Colombia; 4 Oncology Unit, Clínica Porto Azul, Barranquilla 080001, Colombia; hernandezabraham86@gmail.com; 5 Hospital Serena del Mar, Cartagena 130007, Colombia; 6 Functional Breast Cancer Unit, Centro de Tratamiento e Investigación Sobre el Cáncer Luis Carlos Sarmiento Angulo (CTIC), Bogotá 110131, Colombiapmarc@fctic.org (M.E.P.); hernan_carranza@yahoo.com (H.C.);; 7 GIGA Research Group, Universidad El Bosque, Bogotá 110141, Colombia; 8 Clinical and Applied Cancer Research (FICMAC), Bogotá 110111, Colombia; 9 Colsanitas, Bogotá 111321, Colombia; 10 Fundación Santa Fe de Bogotá, Bogotá 110111, Colombia; 11 Clínica del Country, Bogotá 110221, Colombia; 12 Odds Epidemiology, Bogotá 111321, Colombia; gerenciamedica@oddsepidemiology.com (J.R.Z.P.); aurguttrab@gmail.com (A.V.G.R.)

**Keywords:** advanced breast cancer, HER2-positive, HER2-low, targeted therapy, expert consensus, clinical recommendations, Colombia

## Abstract

**Background/Objectives:** The management of advanced HER2-positive and HER2-low breast cancer is evolving rapidly. This study aimed to generate national, evidence-based clinical recommendations for Colombia, addressing the lack of standardized local guidance. **Methods:** A systematic literature search (2020–2024) was conducted. The evidence was evaluated using AGREE II, RoB 2, ROBINS-I, and JBI tools. Recommendations were developed using the GRADE approach and a structured modified Delphi consensus process involving 34 national experts. **Results:** A total of 22 clinical recommendations were formulated. These address HER2 quantification and reporting, first-line and subsequent-line therapy, and management of brain metastases. Five recommendations (23%) were classified as strong, supported by high-certainty evidence, while 17 (77%) were conditional, reflecting the need for adaptation to local contexts and resource availability. **Conclusions:** This consensus provides updated, context-specific clinical guidance to optimize the care of patients with advanced HER2-positive and HER2-low breast cancer in Colombia. The recommendations are designed to standardize care, improve patient outcomes, and promote equitable access to innovative therapies within the Colombian healthcare system.

## 1. Introduction

Breast cancer (BC) is the most common neoplasm in women globally and ranks as the second most frequent cancer when considering both sexes. According to recent data from GLOBOCAN 2022, 2,296,840 new cases were reported, representing 11.5% of all cancers and 23.8% of cancers in women. Moreover, it caused 666,103 deaths, making it the fourth leading cause of cancer-related mortality worldwide. The five-year prevalence reached 8.17 million cases, highlighting its significant global public health burden [1].

In Colombia, BC is also the most frequent neoplasm in women, with an estimated 17,018 new cases in 2022, accounting for 27.7% of female cancer diagnoses. During the same period, annual mortality from this cause was 4752 deaths, positioning it as the fourth leading cause of cancer-related death in the country and the first among women [1].

Advances in molecular biology have enabled the identification of breast cancer subtypes with therapeutic relevance, particularly those defined by the expression of the human epidermal growth factor receptor 2 (HER2). It is estimated that 20–25% of cases are HER2-positive, and among these, approximately half also express hormone receptors [2]. Additionally, a newly described subtype known as HER2-low, characterized by HER2 IHC 1+ or 2+ with a negative in situ hybridization (ISH) result, accounts for approximately 50% of all breast cancer cases [3].

The availability of antibody-drug conjugates such as trastuzumab deruxtecan (T-DXd) has demonstrated clinical benefits in patients with advanced HER2-low disease, improving both overall survival and progression-free survival [4]. These advances underscore the need for rigorous and standardized HER2 testing and pathological characterization as recommended by ASCO/CAP guidelines and the ESMO consensus [5,6].

In this context, it is essential to have clinical recommendations adapted to the Colombian healthcare system to guide accurate diagnosis, molecular classification, and selection of targeted therapies. This national consensus, based on evidence and formal expert consensus, aims to optimize the care of patients with advanced HER2-positive and HER2-low breast cancer, improving clinical outcomes and promoting equitable access to innovative therapies.

## 2. Materials and Methods

### 2.1. Methodological Design

This clinical consensus was developed through a systematic and rigorous approach, integrating the best available scientific evidence and a formal consensus process using the modified Delphi method [7,8,9]. The objective was to formulate clinical recommendations contextualized to the Colombian healthcare system, based on up-to-date evidence and the experience of national experts in the management of advanced HER2 breast cancer.

### 2.2. Evidence Search and Selection

A systematic literature search was conducted in electronic databases (MEDLINE/PubMed, Embase, ClinicalKey, Scopus, and LILACS), complemented by manual searches in guideline repositories such as GIN, SIGN, NICE, and NCCN. The search covered publications from January 2020 to October 2024, with no language restrictions, prioritizing clinical practice guidelines, formal consensus documents, systematic reviews, and relevant primary studies on advanced breast cancer with HER2 expression [10,11,12,13,14,15].

Search strategies included controlled vocabulary (MeSH, Emtree) and free-text terms related to breast cancer, metastasis, recurrence, and HER2 expression, as well as filters by publication type. Full details of the search strategies are documented in Appendix A.

The systematic review component of this work was conducted and reported in accordance with the PRISMA 2020 guidelines. The completed PRISMA 2020 Checklist is provided as Supplementary Materials.

The systematic review component of this consensus was not registered, as it was performed to inform a formal expert consensus process rather than as an independent systematic review.

A descriptive summary of the key characteristics of the included evidence sources, including study type, geographic scope, target population, clinical setting, and main outcomes, is provided in Appendix B.

### 2.3. Eligibility Criteria

Publications that addressed the guiding questions of the consensus were included, with emphasis on clinical practice guidelines, consensus statements, systematic reviews, and clinically applicable primary studies. Narrative reviews, editorials, conference abstracts, and literature without methodological support or peer review were excluded.

### 2.4. Quality and Certainty Assessment of the Evidence

Methodological quality was assessed using standardized instruments according to the type of study: AGREE II for clinical guidelines [16,17], RoB 2 for randomized controlled trials [18], ROBINS-I [19], and Joanna Briggs Institute (JBI) [20] tools for non-randomized studies and formal consensus statements. Certainty of the evidence was graded according to GRADE as high, moderate, low, or very low [21]. Two reviewers independently extracted data using a standardized data extraction form. Any discrepancies were resolved through discussion or by consulting a third reviewer.

### 2.5. Consensus Process

The consensus was developed through a structured methodology based on the modified Delphi model [9], aiming to produce clinically relevant, feasible, and context-adapted recommendations for Colombia. A total of 34 national experts participated, including oncologists, pathologists, radiation oncologists, surgeons, internists, and other specialists, all with experience in advanced HER2 breast cancer (see Appendix C for the list of participants). Deliberations were conducted through virtual sessions and a final in-person meeting.

A detailed description of the expert panel composition, evidence preparation, and the modified Delphi consensus process is provided in Appendix D.

Each recommendation was approved by open voting using a 9-point Likert scale (1 = total disagreement; 9 = total agreement). Mean, median, and 95% confidence intervals (95% CI) of the scores were calculated. The following decision rules were applied:

- Include recommendation: if at least 80% of experts voted between 7 and 9, or if the median fell within that range.
- Exclude recommendation: if at least 80% voted between 1 and 3, or if the median was within that range.
- Further review: if no clear consensus was reached, additional group discussion and a second vote were conducted.

The results of the voting process and corresponding metrics are presented in Appendix E.

### 2.6. Strength of Recommendations

The classification of recommendations as strong or conditional was determined using the GRADE Evidence-to-Decision (EtD) framework, considering the following domains: priority of the problem, balance between benefits and risks, certainty of the evidence, resource use, feasibility, acceptability, and impact on equity [21].

Criteria for recommendation strength:

- Strong: if ≥95% of experts voted “yes” or “probably yes” in key domains (net benefit, high certainty, feasibility).
- Conditional: if that threshold was not reached or relevant uncertainty existed (low certainty, uncertain balance, or feasibility limitations).

This procedure ensured that all recommendations resulted from a transparent and systematic process, aligned with international standards [22], and adapted for implementation in resource-limited health systems such as Colombia’s.

## 3. Results

### 3.1. Evidence Search and Selection

The systematic search yielded a total of 644 references. After removing 115 duplicates, 529 titles and abstracts were screened, with 30 excluded for irrelevance. A total of 499 publications were assessed using the Rayyan^®^ platform, from which 46 studies were preselected for full-text evaluation. After applying the inclusion criteria, 17 were excluded, and 29 studies were included as direct evidence for the formulation of recommendations.

The direct evidence consisted of: 10 clinical practice guidelines (CPGs) [5,10,11,12,13,14,15,23,24,25], 1 formal ESMO consensus on HER2-low [6], 13 phase II–III clinical trials [4,26,27,28,29,30,31,32,33,34,35,36,37], 4 non-randomized studies [38,39,40,41], and 1 real-world observational study [42]. Additionally, 27 supplementary references were used for contextualization (citation chaining), without affecting the primary synthesis. The PRISMA diagram detailing the entire process is presented in Appendix F.

### 3.2. Assessment of Methodological Quality

The 10 clinical guidelines were assessed using AGREE II, with high methodological quality observed (≥60% score in rigor and clarity domains) [5,10,11,12,13,14,15,23,24,25]. The ESMO consensus was assessed with the JBI tool for formal guidelines, meeting all criteria for transparency, clinical expertise, conflict of interest management, and evidence-based formulation [6].

The 13 clinical trials were evaluated using RoB 2. Eight trials (e.g., DESTINY-Breast03/04, HER2CLIMB) showed low risk of bias [4,26,27,29,32,33,34,37], four raised some concerns [28,30,31,36], and one (LANTERN) was considered high risk due to methodological limitations [35].

The 4 non-randomized studies were evaluated with JBI tools. PERUSE and KAMILLA were rated as moderate quality [38,39], while DAISY and DESTINY-Breast01 were considered moderate to high quality despite the absence of a control group [40,41]. The observational study by Petit et al. was rated as having a serious risk of bias with ROBINS-I, due to uncontrolled confounding and missing data [42].

### 3.3. Certainty of the Evidence (GRADE)

The certainty of the evidence for each key question was assessed using the GRADE framework and is summarized in Appendix G. Certainty was classified as follows:

- High for recommendations supported by phase III RCTs with low risk of bias (e.g., CLEOPATRA, DESTINY-Breast03) [26,29,43].
- Moderate in contexts with clinical heterogeneity or limited precision (e.g., third-line treatment) [27,34,37].

This was not applicable for recommendations based solely on high-quality clinical guidelines and formal consensus documents, where GRADE was not applied, but inclusion was justified by methodological robustness [5,6,10].

### 3.4. Consensus Results and Strength of Recommendations

A total of 22 clinical recommendations were formulated. After group discussion and open voting:

- 17 recommendations (77%) were classified as conditional, due to low-to-moderate certainty or local feasibility constraints.
- 5 recommendations (23%) were classified as strong, with high certainty and immediate applicability.

Voting achieved ≥80% agreement in all cases. For conditional recommendations, the need for adaptation based on available resources and biomarker access was explicitly justified.

### 3.5. Clinical Recommendations

#### 3.5.1. Question 1: Criteria for Quantifying and Reporting HER2 Expression

Evaluation of HER2 status is essential to determine eligibility for targeted therapies in advanced breast cancer. Direct evidence comes from high-quality international guidelines (ASCO/CAP 2018, ASCO/CAP 2023, ESMO 2023) [5,6,44], assessed using AGREE II. A recent formal consensus specifically addressing HER2-low was also included [6]. No primary studies were identified that modify current standards for HER2 quantification or reporting. The overall certainty of the evidence was considered high, due to methodological robustness, consistency across sources, and direct clinical applicability.

Guidelines recommend mandatory use of immunohistochemistry (IHC) with standardized scoring (0, 1+, 2+, 3+), complemented by in situ hybridization (ISH) to confirm HER2 amplification in IHC 2+ cases. Detailed reporting is essential to identify patients eligible for antibody-drug conjugates such as T-DXd, particularly in HER2-low and HER2-ultralow contexts [4]. Appropriate interpretation requires validated assays and adherence to international diagnostic algorithms.

##### Recommendations

1. At the time of initial diagnosis of advanced breast cancer—whether newly diagnosed or recurrent—it is recommended to perform a biopsy of the primary tumor or metastatic lesion to assess estrogen receptor, progesterone receptor, and human epidermal growth factor receptor 2 (HER2) status (expert recommendation).
  Certainty of the evidence: high; strong recommendation.
2. In patients with recurrent metastatic disease, HER2 status should be evaluated using tissue from the metastatic site, provided that such a sample is available (expert recommendation).
  Certainty of the evidence: high; strong recommendation.
3. When reporting HER2 test results, pathologists should use nomenclature aligned with the ASCO/CAP 2018 and ESMO 2023 guidelines. Reports must include the HER2 immunohistochemistry (IHC) score (0, 1+, 2+, or 3+) to support the identification of patients with HER2-low or ultra-low expression (expert recommendation).
  Certainty of the evidence: high; strong recommendation.
4. A validated HER2 test, consistent with current international guidelines, should be used to identify HER2 0, 1+, or HER2 2+/ISH non-amplified breast cancer. Appropriate assays for detecting HER2 protein include the Pathway 4B5 assay (used in the DB-04 study) or any other validated test deemed suitable by pathologists, based on the available evidence and practical considerations (expert recommendation).
  Certainty of the evidence: moderate; conditional recommendation.

#### 3.5.2. Question 2: First-Line Treatment for Metastatic HER2-Positive Breast Cancer

First-line treatment for advanced HER2-positive breast cancer should optimize progression-free survival (PFS) and overall survival (OS), considering tolerability and access to targeted therapies. Direct evidence comes from high-quality phase III trials, such as CLEOPATRA, which demonstrated significant benefits with the combination of trastuzumab, pertuzumab, and docetaxel [43]. Additionally, studies such as PHILA and DESTINY-Breast03 provide evidence for the use of T-DXd in early relapses [26,32].

International guidelines support the combination of trastuzumab, pertuzumab, and a taxane as standard, followed by maintenance with the antibodies. However, clinical scenarios where this regimen is not feasible were identified, including chemotherapy contraindications, comorbidities, and early relapse after adjuvant treatment. In these contexts, evidence supports T-DXd as an alternative, and combinations with eribulin or endocrine therapy plus anti-HER2 according to hormone receptor status [14,28,45]. The overall certainty of evidence was high for the standard regimen and moderate for alternatives. All recommendations were classified as conditional due to the need for clinical adaptation.

##### Recommendations

1. The combination of trastuzumab, pertuzumab, and a taxane is recommended as first-line treatment for patients with HER2-positive breast cancer, for up to six cycles of taxane therapy, unless contraindicated (expert recommendation).
  Certainty of the evidence: High. Recommendation: Conditional
2. First-line treatment for metastatic HER2-positive breast cancer should include pertuzumab, trastuzumab, and docetaxel for up to six cycles, provided it is well tolerated. Maintenance therapy with pertuzumab and trastuzumab should then continue until disease progression, regardless of hormone receptor (HoR) status (expert recommendation).
  Certainty of the evidence: High. Recommendation: Conditional
3. T-DXd is recommended as first-line treatment for patients who experience recurrence within six months of completing adjuvant or neoadjuvant therapy, or for those with contraindications to T-DM1 (Expert recommendation).
  Certainty of the evidence: High. Recommendation: Conditional
4. The combination of eribulin, trastuzumab, and pertuzumab, followed by maintenance with trastuzumab and pertuzumab until disease progression or unacceptable toxicity, may be considered a valid alternative to the standard regimen of taxane, trastuzumab, and pertuzumab in patients with metastatic HER2-positive breast cancer (expert recommendation).
  Certainty of the evidence: Moderate. Recommendation: Conditional
5. In patients with HER2-positive and HoR-negative breast cancer for whom chemotherapy is contraindicated, a HER2-targeted therapy strategy using trastuzumab plus pertuzumab is suggested. If taxanes are specifically contraindicated, another chemotherapy agent may be considered (expert recommendation).
  Certainty of the evidence: Moderate. Recommendation: Conditional
6. In patients with HER2-positive and HoR-positive breast cancer in whom comorbidities, personal preferences, or functional status limit the use of chemotherapy, endocrine therapy—such as an aromatase inhibitor—combined with HER2-targeted therapy (trastuzumab plus pertuzumab) is suggested (expert recommendation).
  Certainty of the evidence: Moderate. Recommendation: Conditional

#### 3.5.3. Question 3: Treatment After Disease Progression in Metastatic HER2-Positive Breast Cancer

Disease progression in HER2-positive breast cancer requires effective therapies in second-line and beyond. Direct evidence is mainly from high-quality phase III trials. The DESTINY-Breast03 trial demonstrated the superiority of T-DXd over T-DM1 in second-line, significantly improving progression-free and overall survival [26]. These findings led to T-DXd being positioned as the preferred treatment in this line.

In later lines, options include T-DM1, tucatinib combined with trastuzumab and capecitabine (HER2CLIMB) [27], and various anti-HER2-based regimens. However, evidence for third-line and beyond comes from studies with lower certainty, including post hoc analyses, observational studies, and clinical experience [30,38,39,40]. Overall certainty was high in second-line and moderate to low in later lines, supporting the classification of most recommendations as conditional.

##### Recommendations

1. T-DXd is recommended as the preferred second-line treatment option for patients with HER2-positive breast cancer who experience disease progression after receiving a trastuzumab-based regimen (expert recommendation).
  Certainty of the evidence: High. Recommendation: Strong
2. T-DXd is recommended as second-line therapy in patients with metastatic HER2-positive breast cancer who have progressed during or after treatment with trastuzumab, pertuzumab, and chemotherapy (expert recommendation).
  Certainty of the evidence: High. Recommendation: Strong
3. T-DXd is recommended for patients with advanced HER2-positive breast cancer who have previously received trastuzumab, pertuzumab, and T-DM1 (expert recommendation).
  Certainty of the evidence: High. Recommendation: Conditional
4. Continued anti-HER2 therapy beyond the second line is suggested in patients with metastatic breast cancer, as clinical benefit persists in third-line and later-line settings (expert recommendation).
  Certainty of the evidence: Moderate. Recommendation: Conditional
5. For patients previously treated with T-DXd, the choice of subsequent treatment regimens, routes of administration, and toxicity management should be individualized through a shared decision-making process between the patient and medical team, as no direct comparative studies support a specific regimen (expert recommendation).
  Certainty of the evidence: Low. Recommendation: Conditional
6. Options include the following:
  ○ If the patient has not previously received trastuzumab emtansine (T-DM1) in the second line, a regimen including T-DM1 should be considered.
    Certainty of the evidence: High. Recommendation: Conditional
  ○ Tucatinib in combination with trastuzumab and capecitabine may be offered as another treatment option for advanced disease.
    Certainty of the evidence: High. Recommendation: Conditional
  ○ Neratinib combined with capecitabine may be offered.
    Certainty of the evidence: Low. Recommendation: Conditional
  ○ Lapatinib plus trastuzumab may be offered.
    Certainty of the evidence: Low. Recommendation: Conditional
  ○ Lapatinib plus capecitabine may be offered.
    Certainty of the evidence: High. Recommendation: Conditional
  ○ Other chemotherapy and trastuzumab combinations may be considered—alternative regimens include the following:
    Certainty of the evidence: Moderate. Recommendation: Conditional
    ◾ Vinorelbine + trastuzumab
    ◾ Capecitabine + trastuzumab
    ◾ Gemcitabine + trastuzumab
    ◾ Eribulin + trastuzumab
    ◾ Carboplatin + trastuzumab
    ◾ Ixabepilone + trastuzumab
    ◾ Liposomal doxorubicin + trastuzumab
  ○ Margetuximab plus chemotherapy may be offered.
    Certainty of the evidence: Moderate. Recommendation: Conditional
  ○ Hormonal therapy plus trastuzumab may be considered (for patients with estrogen receptor-positive [ER+] and/or progesterone receptor-positive [PgR+] disease).
    Certainty of the evidence: Moderate. Recommendation: Conditional
  ○ Abemaciclib combined with trastuzumab and fulvestrant may be offered.
    Certainty of the evidence: Moderate. Recommendation: Conditional
7. Trastuzumab emtansine (T-DM1) should be considered as a second-line treatment option for patients with HER2-positive breast cancer who have progressed after prior treatment with a taxane and trastuzumab, in settings where T-DXd is contraindicated (expert recommendation).
  Certainty of the evidence: High. Recommendation: Conditional
8. The combination of tucatinib, capecitabine, and trastuzumab, as well as T-DXd, should be considered as second-line treatment options for selected patients with HER2-positive breast cancer and brain metastases (expert recommendation).
  Certainty of the evidence: Moderate. Recommendation: Conditional

#### 3.5.4. Question 4: Treatment of Metastatic HER2-Low Breast Cancer

HER2-low is defined as IHC 1+ or 2+ with negative ISH, and represents an emerging subtype with specific therapeutic implications. The strongest evidence comes from the DESTINY-Breast04 trial, which showed that T-DXd significantly improves progression-free and overall survival in patients with HER2-low disease who had received prior chemotherapy and were refractory to endocrine therapy [4]. The certainty of evidence was high.

For HER2-low patients with hormone receptor-negative status, sacituzumab govitecan is a valid alternative, although with less robust comparative evidence. No direct trials compare T-DXd vs. sacituzumab govitecan. Selection is based on indirect analyses, clinical experience, and local availability [40,41,42]. Certainty of evidence was high for T-DXd in HER2-low HR+ population, and moderate for sacituzumab in HER2-low HR–. Recommendations were classified as strong or conditional based on certainty and applicability.

##### Recommendations

1. Patients with metastatic HER2-low breast cancer (defined as IHC 1+ or IHC 2+/ISH-negative) who are hormone receptor-positive, have previously received cyclin-dependent kinase 4/6 (CDK4/6) inhibitors and at least one line of chemotherapy (or who experienced disease progression within six months after [neo] adjuvant chemotherapy), and are considered refractory to endocrine therapy, should be considered candidates for treatment with T-DXd (expert-adapted recommendation).
  Certainty of the evidence: Moderate. Recommendation: Conditional
2. In patients with metastatic HER2-low breast cancer where both T-DXd and sacituzumab govitecan are available, T-DXd is preferred (expert-adapted recommendation).
  Certainty of the evidence: High. Recommendation: Strong
3. Sacituzumab govitecan is suggested over T-DXd in patients with metastatic HER2-low breast cancer who are hormone receptor-negative (expert-adapted recommendation).

Certainty of the evidence: Moderate. Recommendation: Conditional

#### 3.5.5. Question 5: Treatment of CNS Progression in HER2-Positive and HER2-Low Breast Cancer

Central nervous system (CNS) metastases are common in HER2-positive breast cancer, requiring integrated strategies. Direct evidence comes from the HER2CLIMB study, which demonstrated the efficacy of tucatinib, trastuzumab, and capecitabine in patients with brain metastases, improving PFS and OS, including in active CNS disease [27]. Observational evidence from KAMILLA also supports CNS efficacy of T-DXd, though with lower methodological strength [39].

In HER2-low, no specific trials addressed brain metastases, but indirect findings suggest T-DXd may be considered due to CNS penetration and activity. Certainty of evidence was high for tucatinib in HER2-positive and moderate for T-DXd in HER2-positive and HER2-low. Due to clinical heterogeneity and the need for individualized decisions, both recommendations were classified as conditional.

##### Recommendations:

1. In patients with metastatic HER2-positive breast cancer who have achieved local control of the disease and have no evidence of systemic progression, it is suggested to consider continuing the previously established systemic treatment (expert consensus-based recommendation).
  Certainty of the evidence: Low. Recommendation: Conditional
2. In patients for whom a change in systemic treatment is being considered:
  Certainty of the evidence: Moderate. Recommendation: Conditional
  ○ For HER2-positive disease:
    ◾ The combination of tucatinib, capecitabine, and trastuzumab is the preferred regimen, particularly in oligosymptomatic patients with limited disease.
    ◾ T-DXd may also be considered.
  ○ For HER2-low disease:
    ◾ T-DXd may also be considered

Certainty of the evidence: Moderate. Recommendation: Conditional.

## 4. Discussion

This evidence-based clinical consensus provides updated guidelines for the diagnosis and treatment of advanced HER2-positive and HER2-low breast cancer in Colombia. The recommendations reflect the best available scientific evidence, contextualized through a structured consensus process involving national experts. Most recommendations were classified as conditional, reflecting both the need for adaptation to local settings and the clinical heterogeneity of the target population.

Therapeutic advances in HER2-positive breast cancer, such as the combination of trastuzumab, pertuzumab, and chemotherapy, have established a standard of care with a high level of certainty [14,43]. However, early relapses or contraindications to chemotherapy pose challenges in clinical practice, where alternatives like T-DXd have demonstrated efficacy in recent studies [26]. Likewise, the definition and management of the HER2-low subtype represent a paradigm shift in breast oncology. The DESTINY-Breast04 trial showed that patients with low HER2 expression can benefit from targeted therapies, expanding the available treatment options [4].

Implementation of these strategies in Colombia faces barriers related to access to precise diagnostic technologies, availability of high-cost therapies, and inequalities within the healthcare system. Therefore, this consensus emphasizes the need for detailed HER2 reporting and rational selection of validated tests, in accordance with international standards [5,6]. Moreover, clinical decision-making must consider feasibility, acceptability, and efficient use of resources, aligned with the GRADE and equity frameworks [46].

It is important to highlight that, although high-quality sources were used, not all clinical scenarios are supported by direct evidence. In such cases, recommendations were formulated based on prior consensus documents, observational data, and national clinical experience [38,39,40]. The consensus process ensured transparency and representativeness, with a high level of agreement among participants.

This consensus also identifies gaps in the evidence, such as the lack of specific studies on HER2-low with brain metastases, direct comparisons between antibody–drug conjugates, and the efficacy of combinations in specific subgroups. These issues should be addressed in future research involving Latin American populations. Furthermore, as a consensus guideline based on a narrative synthesis of the literature, this review does not provide the quantitative estimates of treatment effects that a formal meta-analysis would yield.

## 5. Conclusions

This consensus provides context-specific clinical recommendations for the management of advanced HER2-positive and HER2-low breast cancer, based on updated evidence and a formal expert consensus process. The recommendations are aligned with international guidelines but adapted to the realities of the Colombian healthcare system. Their implementation aims to optimize clinical decision-making, improve patient outcomes, and promote equitable access to innovative therapies.

This document serves as a reference tool for healthcare professionals, institutions, and decision-makers, aimed at standardizing care and reducing inequities in the treatment of advanced breast cancer in the country.

## Figures and Tables

**Figure A1:**
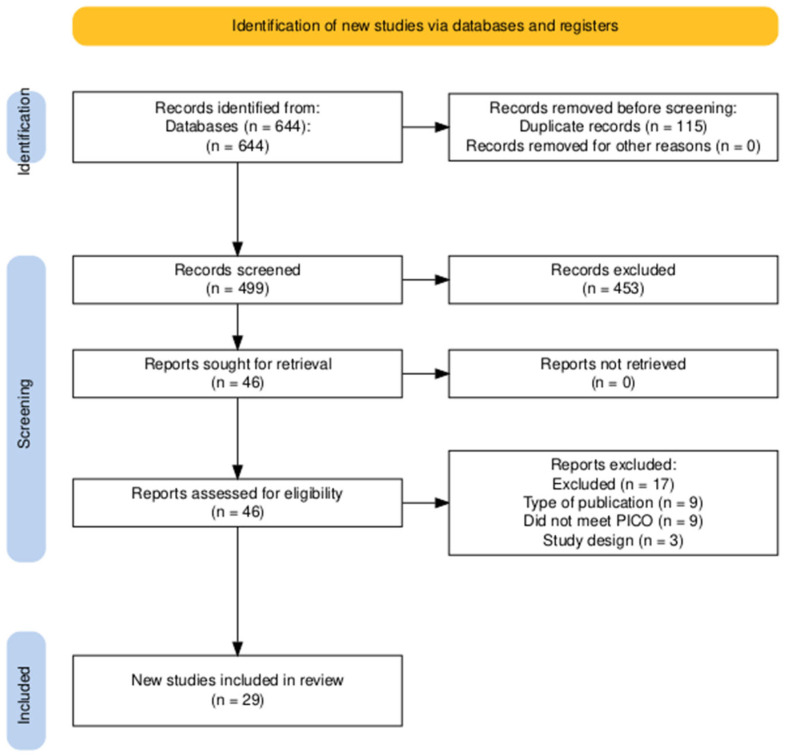
PRISMA Flow Diagram.

## Data Availability

No new data were generated or analyzed in this study. All data supporting the findings of this work are contained within the article and its appendices.

## Supplementary Materials

The following supporting information can be downloaded at: https://www.mdpi.com/article/10.3390/jcm15020514/s1, PRISMA 2020 Checklist [47].

## Appendix A. Sources and Search Strategies

The following tables detail the search strategies applied across various databases to identify clinical practice guidelines and evidence for HER2-positive and HER2-low advanced breast cancer.

**Table A1:** MEDLINE/PubMed search strategy.

Feature	Search Terms	Results
Database	Medline	313
Platform	PubMed	
Search date	October 2024	
Date range	Last 5 years	
Language restrictions	None	
Other limits	Clinical practice guidelines	
Search strategy 1	“breast neoplasms” [MeSH Terms] OR (“Breast” [All Fields] AND “Neoplasms” [All Fields]) OR “breast neoplasms” [All Fields] OR “breast neoplasms” [Title/Abstract] OR “neoplasm breast” [Title/Abstract] OR “breast cancer” [Title/Abstract] OR “cancer breast” [Title/Abstract] OR “cancer of breast” [Title/Abstract] OR “malignant neoplasm of breast” [Title/Abstract] OR “breast malignant neoplasm” [Title/Abstract] OR “breast malignant neoplasms” [Title/Abstract] OR “mammary cancer” [Title/Abstract] OR “breast carcinoma” [Title/Abstract]	510,398
Search strategy 2	“neoplasm metastasis” [MeSH Terms] OR (“Neoplasm” [All Fields] AND “metastasis” [All Fields]) OR “neoplasm metastasis” [All Fields] OR “metastase” [Title/Abstract] OR “metastases” [Title/Abstract] OR “metastasis” [Title/Abstract] OR (“metastasation” [All Fields] OR “metastasic” [All Fields] OR “metastasing” [All Fields] OR “metastasise” [All Fields] OR “metastasised” [All Fields] OR “metastasises” [All Fields] OR “metastasising” [All Fields] OR “metastasization” [All Fields] OR “metastasizes” [All Fields] OR “metastasizing” [All Fields] OR “neoplasm metastasis” [MeSH Terms] OR (“Neoplasm” [All Fields] AND “metastasis” [All Fields]) OR “neoplasm metastasis” [All Fields] OR “metastase” [All Fields] OR “metastases” [All Fields] OR “metastasize” [All Fields] OR “metastasized” [All Fields]) AND “Neoplasm” [Title/Abstract]) OR “metastasis neoplasm” [Title/Abstract] OR “neoplasm metastases” [Title/Abstract]	581,179
Search strategy 3	“recurrance” [All Fields] OR “recurrence” [MeSH Terms] OR “recurrence” [All Fields] OR “recurrences” [All Fields] OR “recurrencies” [All Fields] OR “recurrency” [All Fields] OR “recurrent” [All Fields] OR “recurrently” [All Fields] OR “recurrents” [All Fields] OR “recurrences” [Title/Abstract] OR “relapse” [Title/Abstract] OR “relapses” [Title/Abstract]	983,500
Search strategy 4	“advanced breast cancer” [Title/Abstract] OR “advanced stage” [Title/Abstract]	46,337
Search strategy 5	“HER2” [All Fields] OR “her2 neu” [All Fields] OR (“receptor, erbb 2” [MeSH Terms] OR (“receptor” [All Fields] AND “erbb 2” [All Fields]) OR “erbb-2 receptor” [All Fields] OR “erbb2” [All Fields] OR “genes, erbb 2” [MeSH Terms] OR (“genes” [All Fields] AND “erbb 2” [All Fields]) OR “erbb-2 genes” [All Fields]) OR “HER2-positive” [All Fields] OR “HER2-low” [All Fields]	60,875
Search strategy 6	“HER2” [Title/Abstract] OR “HER2-positive” [Title/Abstract] OR “HER2-low” [Title/Abstract]	44,232
Search strategy 7	#2 OR #3 OR #4	1,477,736
Search strategy 8	#5 OR #6	60,875
Search strategy 9	#1 AND #7 AND #8	17,159
Search strategy 10	#9 AND (consensusdevelopmentconference [Filter] OR consensusdevelopmentconferencenih [Filter] OR guideline [Filter] OR practiceguideline [Filter])	44
Search strategy 11	#10 AND (y_5 [Filter])	6
Search strategy 12	“clinical practice” [Title/Abstract] OR “guideline” [Title/Abstract] OR “clinical guidelines” [Title/Abstract] OR “best practice” [Title/Abstract] OR “best practices” [Title/Abstract] OR “standards” [Title/Abstract] OR “consensus” [Title/Abstract] OR “recommendations” [Title/Abstract]	1,048,827
Search strategy 13	#9 AND #12	1052
Search strategy 14	#13 AND (y_5 [Filter])	467
Search strategy 15	#14 NOT (“systematic review” [Publication Type] OR “systematic reviews as topic” [MeSH Terms] OR “systematic review” [All Fields] OR (“review” [Publication Type] OR “review literature as topic” [MeSH Terms] OR “review” [All Fields]))	325
Search strategy 16	#15 AND ((excludepreprints [Filter]) AND (fft [Filter]) AND (English [Filter] OR Spanish [Filter]))	313
**Feature**	**Search Terms**	**Results**
Database	ClinicalKey	37
Platform	Elsevier	
Search date	October 2024	
Date range	Last 5 years	
Language restrictions	None	
Other limits	Clinical practice guidelines	
Search strategy 1	advanced breast cancer filter guidelines AND Last 5 years	14
Search strategy 2	metastatic breast cancer filter guidelines AND Last 5 years	23
**Feature**	**Search Terms**	**Results**
Database	Scopus	61
Platform	Elsevier	
Search date	October 2024	
Date range	Last 5 years	
Language restrictions	None	
Other limits	Clinical practice guidelines	
Search strategy 1	TITLE (metastatic AND breast AND cancer) OR TITLE (advanced AND breast AND cancer) OR TITLE (recurrence AND breast AND cancer)	26,626
Search strategy 2	TITLE (her2) OR KEY (her2) OR TITLE-ABS-KEY (her2 AND positive AND breast AND cancer) OR TITLE-ABS-KEY (her2 AND neu) OR TITLE (her2 AND negative AND breast AND cancer)	34,393
Search strategy 3	(TITLE (metastatic AND breast AND cancer) OR TITLE (advanced AND breast AND cancer) OR TITLE (recurrence AND breast AND cancer)) AND (TITLE (her2) OR KEY (her2) OR TITLE-ABS-KEY (her2 AND positive AND breast AND cancer) OR TITLE-ABS-KEY (her2 AND neu) OR TITLE (her2 AND negative AND breast AND cancer))	4798
Search strategy 4	#3 AND (KEY (guideline) OR KEY (practice AND guidelines AND as AND topic) OR TITLE (clinical AND practice AND guidelines) OR TITLE (guideline *) OR TITLE (consensus) OR TITLE (recommendations) OR ALL (standardization) OR TITLE (guideline *))	206
Search strategy 5	#4 AND PUBYEAR > 2020 AND PUBYEAR < 2025	97
Search strategy 6	#5 AND (LIMIT-TO (LANGUAGE, “English”))	87
Search strategy 7	#6 AND (EXCLUDE (DOCTYPE, “re”) OR EXCLUDE (DOCTYPE, “no”) OR EXCLUDE (DOCTYPE, “cp”) OR EXCLUDE (DOCTYPE, “le”))	61
**Feature**	**Search Terms**	**Results**
Database	LILACS	0
Platform	BVS (Virtual Health Library)	
Search date	October 2024	
Date range	Last 5 years	
Language restrictions	None	
Other limits	Clinical practice guidelines	
Search strategy 1	ti:((cáncer de seno) OR (neoplasia de la mama) OR (carcinoma de mama) OR (tumor de seno))	1161
Search strategy 2	ab:((cáncer de seno) OR (neoplasia de la mama) OR (carcinoma de mama) OR (tumor de seno))	3124
Search strategy 3	ti:(metástasis de la neoplasia) OR ti:(metástasis) OR ti:(recurrencia) OR ti:(recaída) OR ti:(cáncer de mama avanzado)	92,297
Search strategy 4	(her2) OR (her2/neu) OR (erbb2)	141,591
Search strategy 5	#1 AND #2 AND #3 AND #4	5
Search strategy 6	#5 AND (year_cluster:[2019 TO 2024])	3
Search strategy 7	#6 AND clinical practice guidelines	0
**Feature**	**Search Terms**	**Results**
Database	LILACS	124
Platform	BVS (Virtual Health Library)	
Search date	October 2024	
Date range	Last 5 years	
Language restrictions	None	
Other limits	Clinical practice guidelines	
Search strategy 1	ti:((advanced breast cancer) OR (metastatic breast cancer) OR (breast cancer recurrence))	23,539
Search strategy 2	ti:(her2) OR ti:(her2/neu) OR ti:(erbb2)	23,256
Search strategy 3	#1 AND #2	2990
Search strategy 4	#3 AND type_of_study:(“guideline”)	243
Search strategy 5	#4 AND year_cluster: [2019 TO 2024]	125
Search strategy 6	#5 AND language:(“en” OR “es”)	124

Note: indicates the sequential numbering of search sets within the database search strategy. * Indicates truncation and retrieves all words with the same root (e.g., guideline retrieves guideline and guidelines).

**Table A2:** Search Strategies for Clinical Practice Guidelines (CPG) in Repositories.

Date	Source	Search Algorithm	Results	Duplicates	Pre- Selection
Oct-24	GIN	metastatic breast cancer filter 2020 AND 2021 AND 2022 AND 2023 AND 2024	3	0	3
Oct-24	GIN	advanced breast cancer	6	0	0
Oct-24	SIGN	metastatic breast cancer	1	0	0
Oct-24	NICE	metastatic breast cancer AND Last updated date between: 1 January 2020 and 10 September 2024	1	0	1
Oct-24	NICE	advanced breast cancer AND Last updated date between: 1 January 2020 and 10 September 2024	0	0	0
Oct-24	NCCN	Guidelines Breast Cancer Metastatic	33	2	8

## Appendix B

**Table A3:** Characteristics of Studies Included in the Evidence Base for the Consensus.

First Author, Year	Study Type	Geographic Scope	Target Population	Clinical Setting/Line of Therapy	Main Intervention(s)	Comparator(s)	Primary Outcome(s)
**Garcia-Saenz JA et al., 2023**	Clinical Practice Guideline	Spain	Advanced breast cancer	Diagnosis and treatment of advanced disease	Systemic therapies (endocrine, anti-HER2, chemotherapy, etc.)	Not applicable	Consensus recommendations
**Shimoi T et al., 2020**	Clinical Practice Guideline	Japan	Breast cancer patients (systemic treatment)	Systemic treatment (adjuvant, neoadjuvant, metastatic)	Systemic therapies (endocrine, chemotherapy, anti-HER2)	Not applicable	Recommendations, strength of recommendation, strength of evidence
**Terada M et al., 2023**	Clinical Practice Guideline	Japan	Breast cancer patients (systemic treatment)	Early and metastatic breast cancer	Systemic therapies (updates on S-1, abemaciclib, trastuzumab deruxtecan)	Not applicable	Recommendations, strength of recommendation, strength of evidence
**Yamamoto Y et al., 2024**	Clinical Practice Guideline (General Statements)	Japan	Breast cancer patients	Diagnosis, surgery, radiotherapy, systemic therapy	General treatment concepts and algorithms	Not applicable	Clinical recommendations
**Arpino G et al., 2023**	Randomized, open-label, multicenter phase II trial (PERTAIN)	8 countries (71 sites)	HER2-positive, hormone receptor–positive metastatic or locally advanced breast cancer	First-line (previously untreated in metastatic setting)	Pertuzumab + trastuzumab + aromatase inhibitor	Trastuzumab + aromatase inhibitor	Progression-free survival
**Hua X et al., 2022**	Randomized, open-label, non-inferiority phase III trial (SYSUCC-002)	China (9 hospitals)	Hormone receptor–positive, HER2-positive metastatic breast cancer	First-line	Trastuzumab + endocrine therapy	Trastuzumab + chemotherapy	Progression-free survival
**Saura C et al., 2020**	Randomized, active-controlled phase III trial (NALA)	28 countries	Patients with centrally confirmed HER2-positive metastatic breast cancer (MBC) with ≥2 previous HER2-directed MBC regimens	Metastatic setting: previously treated (≥2 previous HER2-directed regimens)	Neratinib + capecitabine	Lapatinib + capecitabine	Centrally confirmed progression-free survival and overall survival
**Murthy RK et al., 2020**	Randomized, double-blind trial (HER2CLIMB)	International (155 sites, 15 countries)	HER2-positive metastatic breast cancer (with or without brain metastases)	Previously treated with trastuzumab, pertuzumab, and trastuzumab emtansine	Tucatinib + trastuzumab + capecitabine	Placebo + trastuzumab + capecitabine	Progression-free survival (first 480 patients)
**Cortés J et al., 2022**	Multicenter, open-label, randomized phase 3 trial (DESTINY-Breast03)	15 countries (169 centers)	HER2-positive metastatic breast cancer	Previously treated with trastuzumab and a taxane	Trastuzumab deruxtecan	Trastuzumab emtansine	Progression-free survival (blind independent central review)
**Bardia A et al., 2024**	Multicenter, open-label, randomized phase 3 trial (DESTINY-Breast06)	Multicenter (324 sites)	Hormone receptor–positive, HER2-low or HER2-ultralow metastatic breast cancer	Metastatic setting; after one or more lines of endocrine-based therapy; no prior chemotherapy for metastatic disease	Trastuzumab deruxtecan	Physician’s choice of chemotherapy	Progression-free survival in the HER2-low population (blind independent central review)
**Gennari A et al., 2021**	Clinical Practice Guideline (ESMO)	Europe	Metastatic breast cancer	Diagnosis, staging, and treatment of metastatic disease	Systemic and locoregional interventions	Not applicable	Evidence-based recommendations
**Tarantino P et al., 2023**	Expert Consensus Statements (ESMO)	International panel (9 countries)	HER2-low breast cancer	Definition, diagnosis, and clinical management	Definitions and therapeutic strategies (e.g., ADCs)	Not applicable	Consensus statements
**Seligmann JF et al., 2020**	Randomized, open-label phase II screening trial (LANTERN)	UK (17 centers)	HER2-positive metastatic breast cancer with CNS metastases	Current or previously treated with trastuzumab; new or recently progressed CNS metastases	Lapatinib + capecitabine	Trastuzumab + capecitabine	Time to progression of CNS metastases
**Al Sukhun S et al., 2024**	Resource-Stratified Guideline (ASCO)	Global (focus on resource-constrained settings)	Metastatic breast cancer in resource-constrained settings	Basic, Limited, and Enhanced settings; first, second, and third lines	Systemic treatments adapted to resource availability	Not applicable	Resource-stratified treatment recommendations
**Rugo HS et al., 2021**	Randomized, open-label phase 3 trial (SOPHIA)	17 countries (166 sites)	ERBB2 (HER2)-positive advanced breast cancer	Progression on $\ge$ 2 prior anti-ERBB2 therapies	Margetuximab + chemotherapy	Trastuzumab + chemotherapy	Progression-free survival (central blinded analysis) and overall survival
**Modi S et al., 2022**	Multicenter, open-label, randomized phase 3 trial (DESTINY-Breast04)	Multicenter	HER2-low metastatic breast cancer	Previously treated with one or two lines of chemotherapy	Trastuzumab deruxtecan	Physician’s choice of chemotherapy	Progression-free survival in the hormone receptor–positive cohort
**Ramakrishna N et al., 2022**	Guideline Update (ASCO)	Not specified (US society guideline)	HER2-positive advanced breast cancer with brain metastases	Management of brain metastases (local and systemic therapy)	Local therapies (surgery, RT) and systemic therapies	Not applicable	Recommendations on local and systemic therapy
**Emens LA et al., 2020**	Randomized, double-blind, placebo-controlled phase 2 trial (KATE2)	9 countries (Asia, Australia, North America, Western Europe)	HER2-positive advanced breast cancer	Previously treated with trastuzumab and a taxane	Trastuzumab emtansine + atezolizumab	Trastuzumab emtansine + placebo	Investigator-assessed progression-free survival
**Tolaney SM et al., 2020**	Randomized, open-label phase 2 trial (monarcHER)	14 countries	Hormone receptor-positive, HER2-positive advanced breast cancer	Advanced setting; previously treated (at least two prior HER2-targeted therapies)	Abemaciclib + trastuzumab + fulvestrant (Group A) and Abemaciclib + trastuzumab (Group B)	Standard-of-care chemotherapy + trastuzumab (Group C)	Investigator-assessed progression-free survival
**Xu B et al., 2021**	Multicenter, open-label, randomized, controlled phase 3 trial (PHOEBE)	China (29 hospitals)	HER2-positive metastatic breast cancer	Previously treated with trastuzumab and taxanes	Pyrotinib + capecitabine	Lapatinib + capecitabine	Progression-free survival (masked independent central review)
**Giordano SH et al., 2022**	Guideline Update (ASCO)	Not specified (US society guideline)	HER2-positive advanced breast cancer	Systemic therapy (first, second, third line+)	HER2-targeted therapies (with/without chemotherapy/endocrine therapy)	Not applicable	Recommendations based on evidence
**Breast Cancer Expert Committee et al., 2024**	Clinical Guideline	China	Advanced breast cancer	Diagnosis and treatment	New therapeutic drugs and treatment models	Not applicable	Recommendations for diagnosis and treatment
**Wolff AC et al., 2023**	Guideline Update (ASCO-CAP)	Not specified (US society guideline)	Patients with breast cancer (for testing)	HER2 testing	HER2 testing algorithms and reporting	Not applicable	Recommendations for HER2 testing and reporting
**Ma F et al., 2023**	Randomized, double-blind, placebo-controlled, multicenter phase 3 trial (PHILA)	China (40 centers)	Untreated HER2-positive metastatic breast cancer	First-line	Pyrotinib + trastuzumab + docetaxel	Placebo + trastuzumab + docetaxel	Progression-free survival assessed by investigator
**Montemurro et al., 2020**	Phase IIIb, international, open-label, single-arm study (KAMILLA),	International	Patients with HER2-positive locally advanced or metastatic breast cancer, specifically analyzing those with baseline brain metastases (BM)	Advanced/metastatic setting; previously treated (prior HER2-targeted therapy and chemotherapy)	Trastuzumab emtansine (T-DM1)	Not applicable (single-arm study)	Best overall response rate and clinical benefit rate (for this exploratory analysis of patients with BM); Safety and efficacy (overall study),
**Miles et al., 2021**	Phase IIIb, global, open-label, single-arm study (PERUSE),	Global (Europe, Asia, North and South America, Africa, and Australia)	Patients with inoperable HER2-positive locally recurrent or metastatic breast cancer (LR/mBC)	First-line therapy for locally recurrent or metastatic breast cancer,	Pertuzumab in combination with trastuzumab and an investigator-selected taxane (docetaxel, paclitaxel, or nab-paclitaxel)	Not applicable (single-arm study)	Safety and tolerability,
**Saura et al., 2023**	Phase II, multicenter, open-label, single-arm trial (DESTINY-Breast01)	Multicenter (Asia, North America, Europe)	Patients with HER2-positive metastatic breast cancer (mBC) resistant or refractory to T-DM1	Metastatic setting; previously treated (resistant or refractory to T-DM1)	Trastuzumab deruxtecan (T-DXd) 5.4 mg/kg	Not applicable (single-arm study)	Confirmed objective response rate (ORR) by independent central review
**Petit et al., 2024**	Multicenter real-world early access program (observational study),	France	Patients with unresectable or metastatic HER2-positive breast cancer	Metastatic setting; previously treated (at least two prior lines of anti-HER2-based regimens),	Trastuzumab deruxtecan (T-DXd)	Not applicable (observational study)	Descriptive analysis of tumor response and safety (no formal primary endpoint reported for this real-world program),
**Mosele et al., 2023**	Phase 2, prospective, open-label clinical trial (DAISY),	France (15 study centers)	Patients with metastatic breast cancer presenting variable HER2 expression (HER2-overexpressing, HER2-low, and HER2 non-expressing)	Metastatic setting; previously treated (at least one line of chemotherapy in the metastatic setting)	Trastuzumab deruxtecan (T-DXd)	Not applicable (multi-cohort single-arm study)	Confirmed objective response rate (ORR),

## Appendix C. Participants in the HER2-Low Consensus

Clinical Experts and Developers

- Abraham Hernández—Oncology
- Álvaro Guerrero—Oncology
- Berlly Díaz—Pathology
- Carlos Calderón—Oncology
- Carlos Vargas—Oncology
- Carolina López Ordoñez—Oncology
- Daniel González—Oncology
- Danilo Aguirre—Oncology
- David Gómez—Radiation Oncology
- Diego Ballén—Oncology
- Diego Gómez—Oncology
- Diego Morán—Oncology
- Fernando Contreras—Oncology
- Fernando Herazo—Mastology
- Henry Idrobo—Oncology
- Hernán Carranza—Oncology
- Juan Carlos Velásquez—Oncology
- Laura Bolaño—Oncology
- Lizbeth Ramírez—Oncology
- Luz F. Sua—Pathology
- Marc Edy Pierre—Oncology
- Marcela Vallejo—Oncology
- María Alejandra Bravo—Oncology
- Mauricio Lema—Oncology
- Maycos Zapata—Oncology
- Milton Lombana—Oncology
- Néstor Llinás—Oncology
- Olga Marcela Urrego—Oncology
- Patricia López—Pathology
- Ricardo Plazas—Oncology
- Sabrina Herrera—Pathology
- Sandra Ximena Franco—Oncology
- Sergio Cervera—Mastology
- William Mantilla—Oncology

Methodological Experts

- Aura Victoria Gutiérrez—Epidemiology
- Julio Zuluaga—Epidemiology
- Juan Pablo Peña—MBA.

## Appendix D. Consensus Development and Modified Delphi Process

Development of the Expert Panel

The consensus was developed by a multidisciplinary national panel composed of 34 clinical experts and three methodological experts. Clinical experts represented multiple relevant specialties involved in the management of advanced breast cancer, including medical oncology, radiation oncology, mastology, and pathology, ensuring a comprehensive and balanced clinical perspective. Methodological experts with formal training in epidemiology and health research methodology supported all phases of the process to ensure rigor, transparency, and methodological consistency.

All participants formally declared potential conflicts of interest and agreed to maintain confidentiality throughout the consensus development process.

Evidence Review and Preparation of Recommendations

A structured review of the literature was conducted before the consensus process, including international clinical practice guidelines and peer-reviewed evidence addressing HER2-positive and HER2-low advanced breast cancer. The methodological team synthesized the available evidence and graded its certainty using the GRADE framework. Based on this synthesis, draft recommendations were formulated to reflect clinically relevant decision points across disease subgroups and treatment scenarios.

Modified Delphi Consensus Process

Consensus development followed a modified Delphi methodology, conducted through four structured virtual panel sessions, each lasting approximately two hours. During these meetings, the methodological team facilitated the discussions and presented synthesized evidence and corresponding draft recommendations, ensuring a standardized process and balanced participation among experts. The expert panel then discussed each recommendation to assess clinical relevance, clarity, and applicability within the Colombian healthcare context.

After the panel discussion, each recommendation was subjected to individual, anonymous voting using an electronic survey (Google Forms^®^). Voting was conducted using a nine-point Likert scale, where scores of 1–3 indicated disagreement, 4–6 indicated relative agreement, and 7–9 indicated total agreement. An a priori consensus threshold of ≥80% agreement among participants was defined.

Quantitative Assessment of Agreement

For each recommendation, quantitative metrics were calculated to objectively assess the robustness of the consensus. These included the total number of votes, mean and standard deviation, median score, 95% confidence interval for the median, and percentage of agreement. These results are reported in detail in Appendix C, allowing full transparency of the voting outcomes.

All five recommendations reached the predefined consensus threshold across the panel sessions, reflecting a high level of agreement among experts.

**Role of Methodological Experts**

Methodological experts oversaw the consensus process, facilitated discussions, ensured adherence to the modified Delphi methodology, and supervised the application of GRADE Evidence-to-Decision principles. They did not vote on clinical recommendations but ensured methodological integrity and consistency in reporting.

## Appendix E

**Table A4:** List of Participating Experts and Affiliations & Consensus Voting Results.

#	Name	Specialty	City	Role
1	Abraham Hernandez	Oncology	Cartagena	Developer
2	Álvaro Guerrero	Oncology	Cali	Clinical Expert
3	Berlly Diaz	Pathology	Bogotá	Clinical Expert
4	Carlos Calderón	Oncology	Bucaramanga	Clinical Expert
5	Carlos Vargas	Oncology	Bogotá	Clinical Expert
6	Carolina López Ordoñez	Oncology	Cali	Clinical Expert
7	Daniel González	Oncology	Medellín	Clinical Expert
8	Danilo Aguirre	Oncology	Valledupar	Clinical Expert
9	David Gómez	Radiotherapy	Medellín	Clinical Expert
10	Diego Ballen	Oncology	Bogotá	Clinical Expert
11	Diego Gómez	Oncology	Bucaramanga	Clinical Expert
12	Diego Moran	Oncology	Medellín	Clinical Expert
13	Fernando Contreras	Oncology	Bogotá	Clinical Expert
14	Fernando Herazo	Mastology	Medellín	Clinical Expert
15	Henry Idrobo	—	—	Clinical Expert
16	Hernán Carranza	Oncology	Bogotá	Developer
17	Juan Carlos Velásquez	Oncology	Bogotá	Clinical Expert
18	Laura Bolaño	Oncology	Barranquilla	Clinical Expert
19	Lizbeth Ramírez	Oncology	Cali	Clinical Expert
20	Luz Sua	Pathology	Cali	Developer
21	Marc Pierre	Oncology	Bogotá	Developer
22	Marcela Vallejo	Oncology	Cali	Clinical Expert
23	Maria Alejandra Bravo	Oncology	Bogotá	Clinical Expert
24	Mauricio Lema	Oncology	Medellín	Developer
25	Maycos Zapata	Oncology	Medellín	Clinical Expert
26	Milton Lombana	Oncology	Cali	Clinical Expert
27	Nestor Llinas	Oncology	Medellín	Clinical Expert
28	Olga Marcela Urrego	Oncology	Cali	Clinical Expert
29	Patricia López	Pathology	Bogotá	Clinical Expert
30	Ricardo Plazas	Oncology	Cúcuta	Clinical Expert
31	Sabrina Herrera	Pathology	Medellín	Clinical Expert
32	Sandra Franco	Oncology	Bogotá	Developer
33	Sergio Cervera	Mastology	Bogotá	Clinical Expert
34	William Mantilla	Oncology	Bogotá	Developer

**Table A5:** Consensus Voting Results.

Metric	Question 1	Question 2	Question 3	Question 4	Question 5
Total votes	32	33	33	29	31
Mean	8.46	7.75	8.6	8.10	8.55
Standard deviation	1.55	1.71	0.56	1.50	0.89
Median	9.0	8.0	9.0	9.0	9.0
95% CI (lower bound)	9.0	7.0	9.0	9.0	9.0
95% CI (upper bound)	9.0	8.0	10.0	10.0	9.0
Consensus (%)	96.43	92.86	100	86.21	96.80
Outcome	Consensus achieved	Consensus achieved	Consensus achieved	Consensus achieved	Consensus achieved

## Appendix G

**Table A6:** Assessment of Certainty of the Evidence (GRADE).

Consensus Question	Type of Included Studies	Risk of Bias	Inconsistency	Indirectness	Imprecision	Publication Bias	Overall Certainty
Q1. HER2 quantification and reporting	CPGs + consensus statements	Not applicable	Not applicable	Not applicable	Not applicable	Not applicable	Not applicable (based on CPGs/consensus)
Q2. First-line treatment for HER2+ metastatic	Phase III RCTs + 1 non-comparative study	Low (RCTs); moderate (non-comparative)	None	Direct	Adequate precision	Not identified	High ⊕⊕⊕⊕
Q3. Treatment after progression in HER2+ metastatic	Phase III RCTs	Low	Low (2L); present (≥3L)	Direct	Adequate precision (2L); lower (≥3L)	Not identified	Moderate ⊕⊕⊕⃝ to High ⊕⊕⊕⊕
Q4. Treatment for HER2-low metastatic	Phase III RCTs	Low	None	Direct	Adequate precision	Not identified	High ⊕⊕⊕⊕
Q5. Treatment after progression in CNS	Phase III RCTs + Phase II + non-comparative studies	Low (RCTs); high (non-randomized)	None (RCTs); present (others)	Direct	Adequate precision (RCTs); lower (others)	Not identified	Low ⊕⊕⃝⃝ to Moderate ⊕⊕⊕⃝

Narrative synthesis/Explanations: The certainty of the evidence was determined based on the analysis of the five GRADE domains: risk of bias, inconsistency, indirectness, imprecision, and publication bias, applied exclusively to direct evidence. In the case of Question 1, certainty was not applicable since recommendations were based on clinical practice guidelines and formal consensus statements evaluated using AGREE II and JBI tools. For the remaining questions, high-quality randomized controlled trials supported high certainty in main scenarios, while certainty was moderate or moderate-low in cases where methodological limitations, imprecision, or heterogeneity of results were identified in specific interventions. Note: Overall certainty of evidence was assessed using the GRADE approach. Symbols indicate certainty levels: ⊕⊕⊕⊕ = high; ⊕⊕⊕⃝ = moderate; ⊕⊕⃝⃝ = low; ⊕⃝⃝⃝ = very low.
